# Retraction: A20 rescues hepatocytes from apoptosis through the NF-κB signaling pathway in rats with acute liver failure

**DOI:** 10.1042/BSR-2018-0316_RET

**Published:** 2021-10-14

**Keywords:** A20, Acute liver failure, Hepatocyte, NF-kB signaling pathway, Tumor necrosis factor α-induced protein 3

This article is being retracted from *Bioscience Reports* at the request of the authors following receipt of a notification from a reader, alerting the Editorial Office to the data in Figures 5A and 5C as well as the Western blots in Figures 1C and 6B.

The Editorial Office contacted the authors to request the raw data and raw images of the Western blots, to which the authors responded with the Retraction request stating that the integrity of the research was damaged. Given the concerns raised the Editor-in-Chief and Editorial Board agree with the Retraction. The co-corresponding author Huang-Shan wishes to take full responsibility for their negligence, and apologizes for the inconvenience caused to the journal and readers.

